# Acute Pulmonary Oedema Secondary to Electrical Cardioversion for Ventricular Tachycardia in a Patient With Severe Left Ventricular Dysfunction

**DOI:** 10.7759/cureus.97965

**Published:** 2025-11-27

**Authors:** Soe Latt Tun, Zaid Qadri, Roshini Kadimcherla, Zay Yar Win, Ingyin May

**Affiliations:** 1 General Medicine, King's College Hospital NHS Foundation Trust, London, GBR

**Keywords:** acute pulmonary oedema, heart failure with reduced ejection fraction, implantable cardioverter-defibrillator, synchronised dc cardioversion, ventricular tachycardia (vt)

## Abstract

Acute pulmonary oedema (APO) is a recognised complication of electrical cardioversion, particularly in patients with significant left ventricular (LV) dysfunction, but is likely underreported in the literature. Awareness of this phenomenon and careful post-procedure monitoring are essential, especially in high-risk individuals.

We report the case of a 76-year-old male with heart failure with reduced ejection fraction (18%), severe mitral and tricuspid regurgitation, a dual-chamber implantable cardioverter-defibrillator (ICD), prior percutaneous coronary intervention, left ventricular thrombus, hypertension, and a renal transplant, who presented with worsening dyspnoea. On admission, he was found to be in monomorphic ventricular tachycardia (VT) at 150 bpm with hypotension (80/46 mmHg). Synchronised cardioversion with 50 J successfully restored sinus rhythm. Fourteen hours later, he developed acute respiratory distress, and chest imaging demonstrated new bilateral perihilar opacities consistent with pulmonary oedema. He was treated with intravenous furosemide, a glyceryl trinitrate infusion, and non-invasive ventilation, resulting in rapid clinical improvement. ICD interrogation confirmed a prolonged VT episode with a therapy threshold set too high for detection, prompting reprogramming of the VT zone to 130 bpm. The patient was discharged in stable condition after six days.

This case underscores that APO after cardioversion may present in a delayed fashion and may be overlooked without prolonged observation. It also highlights the relevance of cardioversion performed for VT, prolonged pre-existing arrhythmic burden, and multiple comorbidities in predisposing patients to post-cardioversion haemodynamic decompensation. Vigilant monitoring and early intervention are crucial to improving outcomes in high-risk patients.

## Introduction

Electrical cardioversion is a widely used and effective intervention for restoring sinus rhythm in both atrial and ventricular arrhythmias, with reported success rates exceeding 85-90% [1]. Although generally well tolerated, it can be followed by clinically significant haemodynamic instability in selected patients. Acute pulmonary oedema (APO) is a recognised complication that appears to be underreported in the literature, particularly in individuals with impaired left ventricular (LV) function or substantial structural heart disease.

The pathophysiological mechanisms underlying APO after cardioversion are incompletely understood and are likely multifactorial. Transient LV systolic dysfunction due to myocardial stunning may reduce contractile performance immediately after electrical shock. The abrupt restoration of coordinated atrial activity can increase LV filling pressures, especially in patients with impaired compliance, significant valvular regurgitation, or pre-existing congestion. Sudden recovery of left atrial mechanical function has been proposed to cause a transient rise in LV end-diastolic pressure, precipitating pulmonary congestion in susceptible individuals [2-4]. Additionally, electrical shock-related disturbances in intracellular calcium handling may contribute to transient diastolic dysfunction and impaired ventricular relaxation, further promoting haemodynamic compromise.

Although APO is most frequently described in patients with LV dysfunction, valvular disease, or renal impairment, cases have also been reported in structurally normal hearts, indicating that cardioversion-related electromechanical changes themselves may play an important role [5]. Observational studies have shown that pulmonary congestion may occur not only immediately after cardioversion but also several hours later, underscoring the dynamic nature of post-procedural haemodynamics and the potential for delayed deterioration [3,6,7].

We describe a case of APO occurring 14 hours after cardioversion for sustained monomorphic ventricular tachycardia in a patient with severe LV dysfunction, advanced valvular regurgitation, renal transplantation, and prolonged pre-admission arrhythmic burden documented on ICD interrogation. This case highlights the potential for delayed cardioversion-related decompensation in high-risk patients and reinforces the need for extended post-procedure monitoring and careful haemodynamic assessment.

## Case presentation

**Figure 1:**
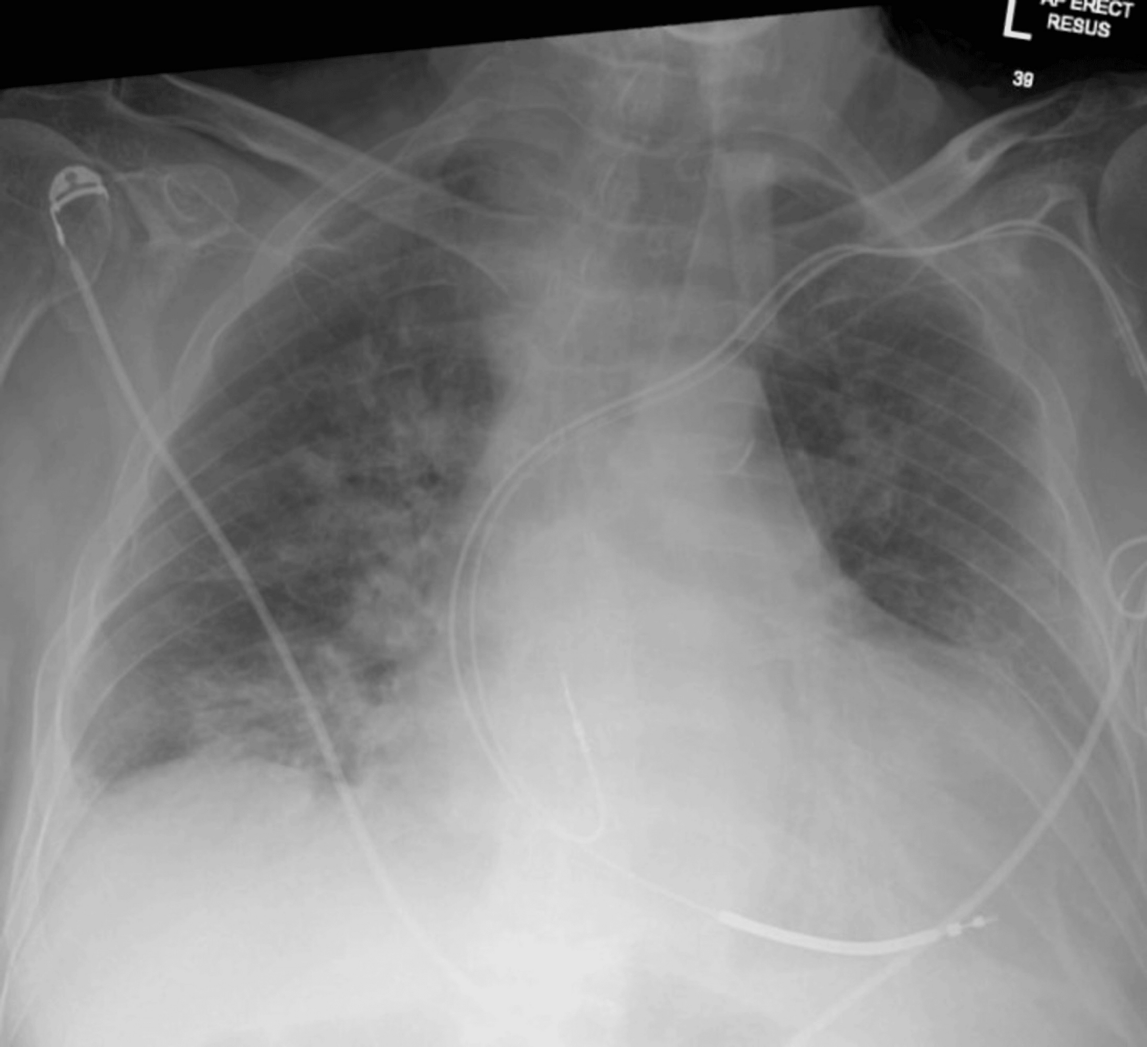
Portable chest X-ray(AP view, semi-erect position) on admission showing pulmonary congestion and cardiomegaly

A 76-year-old male with heart failure with reduced ejection fraction (18%), severe mitral and tricuspid regurgitation, a dual-chamber implantable cardioverter-defibrillator (ICD) implanted for secondary prevention of sudden cardiac death, prior percutaneous coronary intervention, left ventricular thrombus, hypertension, and a renal transplant. He presented with a one-week history of worsening shortness of breath and two days of watery diarrhoea. On examination, he was conscious with a blood pressure of 80/46 mmHg, a palpable pulse, respiratory rate of 22 breaths per minute, and bibasal crackles. His oxygen saturation was unstable, intermittently falling to 87% on room air and requiring frequent titration of supplemental oxygen to maintain saturations above 94%. A chest radiograph showed a right-sided pleural effusion with right-predominant perihilar opacification, consistent with pulmonary congestion, with cardiomegaly unchanged from previous imaging (Figure 1). Early device interrogation indicated that he had been in ventricular tachycardia (VT) for approximately 23 hours prior to arrival, likely contributing to his decompensated state.

**Figure 2:**
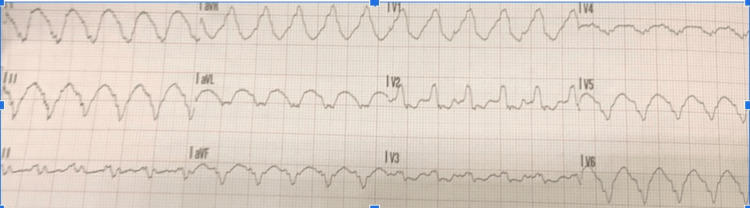
Pre-hospital 12-lead ECG obtained by the ambulance crew demonstrating a wide-complex monomorphic tachycardia (~150 bpm) with markedly prolonged QRS duration (>160 ms) and no visible P waves, consistent with ventricular tachycardia ECG: electrocardiogram

**Figure 3:**
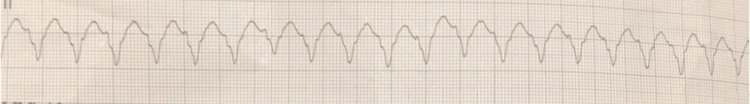
Continuous rhythm strip recorded by the ambulance crew showing sustained monomorphic wide-complex tachycardia at approximately 150–160 bpm, correlating with the sustained VT episode later confirmed on ICD interrogation. VT: ventricular tachycardia; ICD: implantable cardioverter-defibrillator

**Figure 4:**
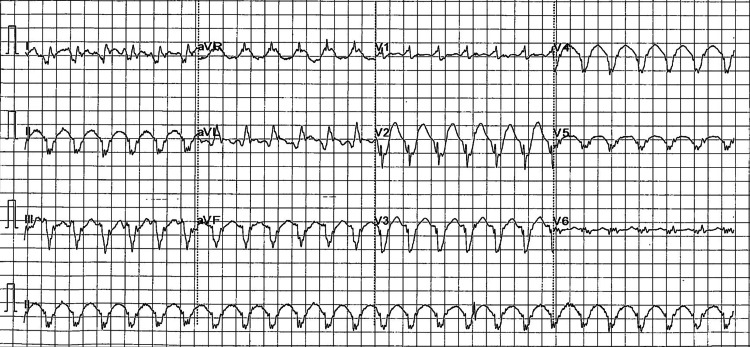
Ventricular tachycardia (VT) on admission (12-lead ECG showing monomorphic VT at 150 bpm) ECG: electrocardiogram

**Figure 5:**
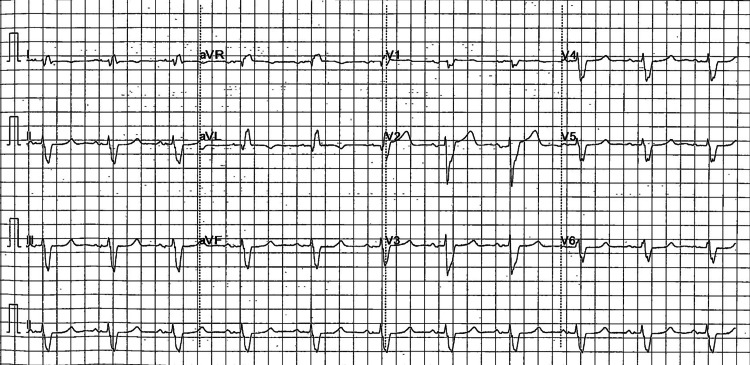
Reversion to sinus rhythm with baseline LBBB following DC cardioversion LBBB: left bundle branch block; DC: direct current.

A pre-hospital 12-lead electrocardiogram (ECG) obtained by the paramedical staff demonstrated a regular wide-complex tachycardia at approximately 150 bpm, consistent with monomorphic VT (Figure 2). A corresponding ambulance rhythm strip demonstrated a sustained wide-complex tachyarrhythmia at the same rate (Figure 3). On arrival to the Emergency Department, the admission ECG again showed a wide-complex tachycardia at 150 bpm (Figure 4). Although the morphology resembled his baseline left bundle branch block (LBBB), the QRS complexes were broader (approximately 160 ms) with no consistent preceding P waves. Continuous cardiac monitoring likewise demonstrated a sustained monomorphic wide-complex tachycardia. Synchronized direct current (DC) cardioversion was performed using a single 50 J shock under intravenous fentanyl and midazolam, successfully restoring sinus rhythm with his baseline LBBB morphology (Figure 5). The VT was considered to have been precipitated by diarrhoea, infection, and acute kidney injury (AKI) on chronic kidney disease (CKD) in a transplanted kidney.

Formal ICD interrogation demonstrated a sustained monomorphic VT at 159 bpm lasting 23 hours and 21 minutes. No ICD therapies were delivered because the VT detection zone was programmed at ≥182 bpm. Lead parameters, sensing, pacing burdens (atrial pacing 6.4%, ventricular pacing 0.1%), and battery longevity (>9.3 years) were satisfactory. Two earlier remote episodes of atrial fibrillation were noted (longest 2 hours 29 minutes, AT/AF burden 0.2%), but these were not temporally related to the presenting arrhythmia. The stored intracardiac electrograms confirmed that the tachycardia present on arrival was ventricular in origin and that this VT terminated immediately following external synchronised cardioversion, providing electrophysiological confirmation that distinguished it from supraventricular tachycardia with aberrancy. In view of his known history of slow VT, the VT detection threshold was reprogrammed from ≥182 bpm to ≥130 bpm with antitachycardia pacing and shock therapies enabled.

Intravenous fluids initiated for AKI were discontinued, and cautious diuresis was commenced, targeting a negative balance of at least 500 mL. Amiodarone and bisoprolol were continued. A transthoracic echocardiogram performed two months earlier showed severe LV systolic dysfunction (EF 18%) and severe mitral and tricuspid regurgitation. As there were no new ischaemic symptoms, no dynamic troponin rise, and no suspicion of new structural abnormality, repeat echocardiography was not expected to alter management and was therefore not repeated. High-sensitivity troponin remained stable (71 → 71 → 74 ng/L). Renal function remained near his CKD baseline with an eGFR of 20-26 mL/min/1.73 m².

**Figure 6:**
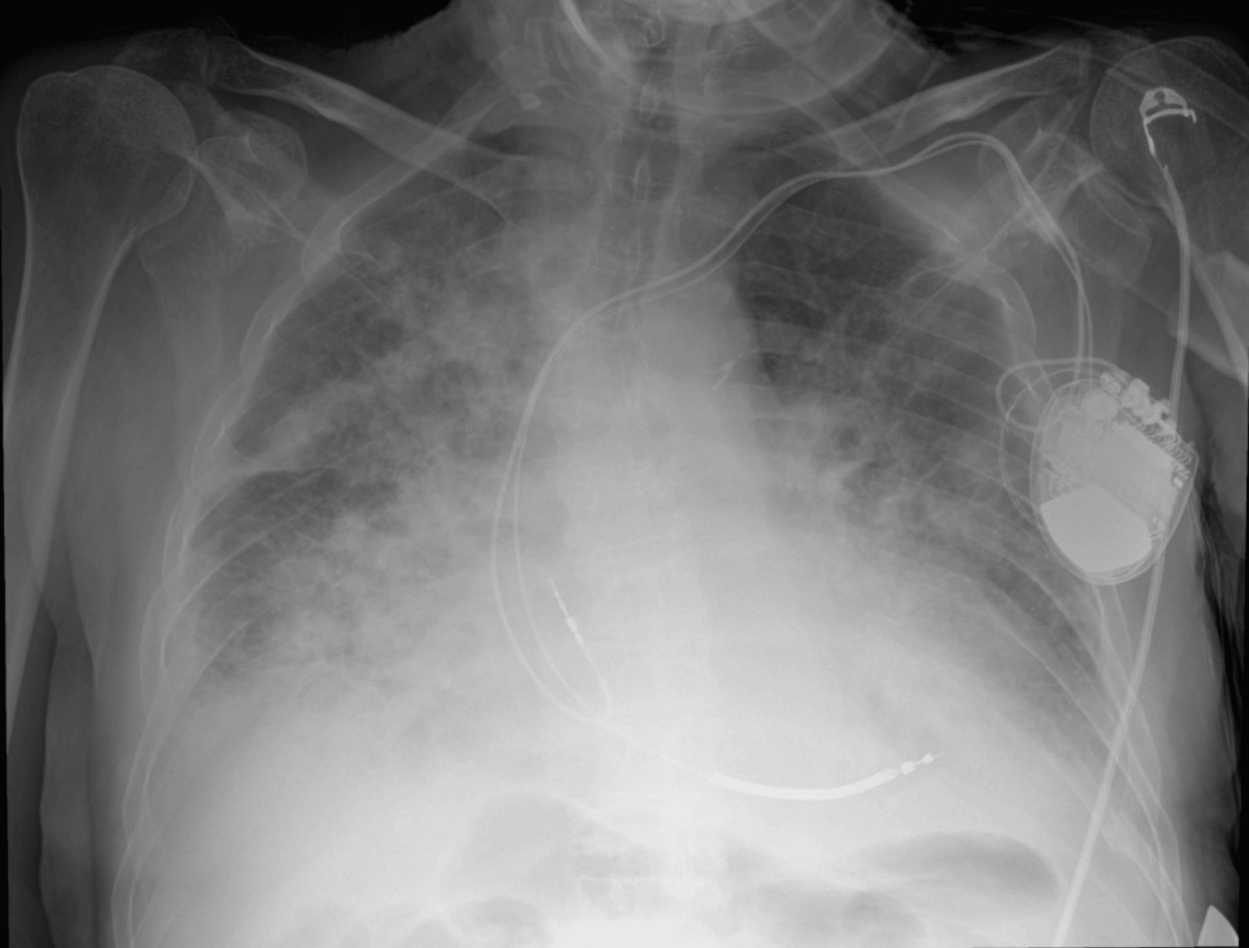
Repeat portable chest X-ray (AP view, sitting position) demonstrating bilateral perihilar opacities consistent with acute pulmonary oedema

Approximately 14 hours after cardioversion, the patient developed acute worsening dyspnoea with new bilateral crackles. His oxygen saturation fell to 83% despite 15 L/min oxygen, and he became markedly tachypnoeic (respiratory rate 40/min) with a blood pressure of 160/75 mmHg and heart rate of 84 bpm. The intensive care team was urgently consulted, and continuous positive airway pressure (CPAP) therapy was initiated, improving oxygen saturation to 92%. An ECG obtained during deterioration showed no new ischaemic changes, although interpretation was limited by respiratory artefact. Repeat chest radiography demonstrated new bilateral perihilar opacities consistent with acute pulmonary oedema (Figure 6). The patient was treated with intravenous furosemide 80 mg, a glyceryl trinitrate infusion titrated to blood pressure, and ventilatory support with CPAP with positive end-expiratory pressure.

His oxygenation and respiratory distress improved within four hours, and CPAP was successfully weaned approximately 24 hours later. He stabilised with optimised medical therapy, including cautious diuresis, continuation of amiodarone and bisoprolol, and close monitoring of renal function and fluid status. He was transferred to the cardiology ward for continued observation and discharged home after six days in stable condition with follow-up arranged through the heart failure team.

Alternative causes of acute pulmonary oedema were systematically considered. Acute coronary syndrome was unlikely, given stable troponin levels, absence of chest pain, and no ischaemic ECG changes. Renal failure was excluded as renal function remained near baseline. There was no fever, leucocytosis, or radiographic evidence of infection (Table 1). Iatrogenic volume overload was also unlikely, as intravenous fluids had been discontinued early after cardiology review, and pulmonary oedema developed 14 hours later. In the absence of other precipitants, the timing and clinical context strongly supported cardioversion-related haemodynamic decompensation as the cause of acute pulmonary oedema.

**Table 1:** Laboratory investigations on admission and at the time of pulmonary oedema MCV: mean corpuscular volume; eGFR CKD-EPI refers to the estimated glomerular filtration rate calculated using the Chronic Kidney Disease Epidemiology Collaboration (CKD-EPI) equation; CRP: C-reactive protein.

Parameters	References	Results on admission	Results after pulmonary oedema
White Cell Count	2.9 - 9.6 10^9^/L	11.8 (High)	13.2 (High)
Haemoglobin	125 - 170 g/L	110 (Low)	94 (Low)
Haematocrit	0.390 - 0.510 L/L	0.352 (Low)	0.298 (Low)
MCV	81 - 100 fL	97	96
Platelet Count	140 - 400 10^9^/L	228	191
Neutrophils	1.50 - 6.10 10^9^/L	8.68 (High)	11.31 (High)
Lymphocytes	0.80 - 3.50 10^9^/L	2.17	0.84
Sodium	135 - 145 mmol/L	140	139
Potassium	3.5 - 5.3 mmol/L	5.3	5.2
Urea	2.5 - 7.8 mmol/L	9.4 (High)	17.4 (High)
Creatinine	61 - 123 µmol/L	274 (High)	242 (High)
eGFR by CKD-EPI (2009)	NA mL/min/1.73m^2^	19 (Low) Baseline 20 - 26	22 (Low)
Adjusted Calcium	2.20 - 2.60 mmol/L	2.41	-
Magnesium	0.70 - 1.00 mmol/L	0.94	-
Phosphate	0.80 - 1.40 mmol/L	1.55 (High)	-
Albumin	35 - 50 g/L	36	-
Alkaline Phosphatase (ALP)	30 - 130 U/L	92	-
Total Bilirubin	21 µmol/L	11	-
Alanine Transaminase	10 - 50 U/L	106	-
CRP	< 5 mg/L	85 (High)	79 (High)
Thyroid-stimulating hormones (TSH)	0.27 - 4.20 mIU/L	12.30 (High)	-
Free Thyroxine (FT4)	11.0 - 21.2 pmol/L	11.6	-
Troponin	<14 ng/L	74 (High) on admission 71 (High) unchanged after 3 hours	74 (High)
NT-proBNP	<400 ng/L	35,000 (High)	32,510 (High)

## Discussion

APO following electrical cardioversion is a recognised but likely underreported complication, most frequently described after cardioversion for atrial fibrillation or flutter. Although often described as rare, its true incidence is probably underestimated, as clinicians who regularly manage patients with structural heart disease frequently anticipate early haemodynamic deterioration and intervene promptly, potentially preventing many cases from being formally documented. As such, transient cardioversion-related haemodynamic instability may be more common than the published literature suggests, particularly in patients with impaired ventricular function [1-4].

The proposed mechanisms of post-cardioversion APO are multifactorial. Transient LV systolic dysfunction due to myocardial stunning may occur immediately after electrical shock, reducing contractile performance for several hours. Restoration of coordinated atrial contraction can abruptly increase LV filling pressures in patients with impaired diastolic compliance or pre-existing congestion. Sudden recovery of left atrial mechanical activity may transiently elevate LV end-diastolic pressure, and electrical shock-induced alterations in intracellular calcium handling may further impair ventricular relaxation [2-4]. These mechanisms are particularly relevant in patients with significant LV dysfunction or valvular regurgitation, as in the present case.

Most published cases describe APO occurring within minutes to a few hours following cardioversion [3]. In contrast, our patient developed acute pulmonary oedema 14 hours after successful cardioversion, representing a delayed presentation. Although delayed cases have been reported, they represent a minority and are clinically important because deterioration outside the immediate post-procedure window may be missed if monitoring is prematurely de-escalated [3]. This case supports recent observational findings that post-cardioversion decompensation can occur several hours after the procedure, particularly in high-risk individuals [6,7].

This case also differs from the majority of previous reports in that cardioversion was performed for sustained monomorphic VT, rather than atrial arrhythmias. APO following VT cardioversion is only rarely reported. Furthermore, ICD interrogation confirmed that the patient had been in VT for more than 23 hours prior to presentation, contributing to haemodynamic stress before cardioversion and likely increasing susceptibility to subsequent pulmonary oedema. The use of ICD-stored intracardiac electrograms, which clearly documented the onset, duration, rate, and termination of VT, provides objective electrophysiological confirmation seldom included in previous case reports and strengthens the causal association between VT cardioversion and subsequent APO.

Alternative causes of acute pulmonary oedema were systematically considered and excluded. Acute coronary syndrome was unlikely: the patient had no chest pain, no ischaemic ECG changes, and no dynamic troponin rise. Acute renal failure did not occur, as renal function remained close to baseline (eGFR 20-26 mL/min/1.73 m²). There was no evidence of infection-no fever, no leucocytosis, and no radiographic features of pneumonia. Intravenous fluids had been discontinued early, and the delayed onset of APO made simple fluid overload unlikely. The temporal association with cardioversion, supported by the absence of other precipitants, therefore strongly favours cardioversion-related haemodynamic decompensation as the underlying mechanism.

The VT episode itself was likely triggered by a combination of diarrhoea, infection, and AKI on CKD. Although electrolyte levels were normal, these conditions can induce sympathetic activation, systemic inflammation, and haemodynamic instability, all of which lower the arrhythmic threshold in patients with ventricular scarring and severe LV dysfunction. This provides a plausible explanation for the prolonged VT documented on ICD interrogation.

Although the admission ECG resembled his baseline left bundle branch block, several elements supported VT over supraventricular tachycardia with aberrancy: the QRS was significantly broader (~160 ms) than baseline; the precordial leads displayed near-negative concordance; there was no consistent relationship between P waves and the QRS complexes; and, most importantly, the ICD electrograms unequivocally demonstrated sustained monomorphic VT at 159 bpm that terminated immediately after external DC cardioversion. This electrophysiological confirmation provides definitive evidence of VT and resolves the diagnostic uncertainty raised by surface ECG interpretation alone.

Treatment of post-cardioversion APO typically includes rapid diuresis, vasodilator therapy, and ventilatory support, with a subset of cases requiring advanced respiratory support [5]. In this case, prompt administration of intravenous furosemide, glyceryl trinitrate infusion, and CPAP resulted in rapid improvement. The coordinated involvement of cardiology, intensive care, and renal teams contributed to the favourable outcome.

This case contributes to the existing literature in several important ways: (1) it documents APO following cardioversion for sustained monomorphic VT, which is far less frequently reported than APO after atrial arrhythmias; (2) it highlights a delayed presentation occurring 14 hours after cardioversion, emphasising the need for extended post-procedural monitoring; (3) it incorporates ICD-confirmed prolonged VT duration as a unique physiological stressor, rarely detailed in previous reports; and (4) it reinforces the importance of considering cardioversion-related haemodynamic changes even when immediate post-shock recovery appears uncomplicated. Awareness of this complication and early intervention are essential to preventing severe respiratory compromise in patients with advanced structural heart disease.

## Conclusions

Acute pulmonary oedema following electrical cardioversion is a recognised complication, particularly among patients with advanced structural heart disease. Although familiar to experienced clinicians, delayed presentations are likely underreported and may be easily overlooked once initial post-cardioversion recovery appears stable. This case illustrates that haemodynamic deterioration can occur many hours after cardioversion, reinforcing the importance of extended observation in high-risk individuals.

A notable feature of this case is that pulmonary oedema developed after cardioversion for sustained monomorphic ventricular tachycardia rather than atrial arrhythmias, which constitute most published reports. In addition, ICD interrogation provided objective evidence of a prolonged VT episode lasting more than 23 hours prior to admission, representing a significant haemodynamic burden. The combination of severe left ventricular systolic dysfunction, significant valvular regurgitation, renal impairment, and sustained tachycardia likely increased the patient’s vulnerability to post-cardioversion decompensation. Early recognition and timely treatment with diuretics, vasodilators, and non-invasive ventilation led to rapid clinical improvement. This case underscores the need for careful peri-procedural assessment, optimisation of volume status, appropriate ICD programming, and vigilant monitoring beyond the immediate post-cardioversion period. It also contributes to the limited literature describing delayed cardioversion-associated pulmonary oedema in the setting of ventricular tachycardia, highlighting a clinically important scenario that may otherwise be underrecognised.
